# Blood flow restriction training in South Africa – a panel discussion

**DOI:** 10.17159/2078-516X/2022/v34i1a14796

**Published:** 2022-01-01

**Authors:** RW Evans, J Ganda, L van Schalkwyk, DL Fabricius, M Cornelissen

**Affiliations:** 1 Enable Centre, Cape Town, South Africa; 2 Sports Rehab Centre, Cape Town, South Africa; 3 Western Province & Stormers Rugby, Cape Town, South Africa; 4 Lambert Sports Clinic, London, United Kingdom; 5 Biokinetics Association of South Africa, Centurion, South Africa

**Keywords:** BFR, occlusion training, HPCSA, biokinetics, physiotherapy

## Abstract

**Background:**

Blood flow restriction (BFR) training uses a cuff to partially occlude venous blood flow and improve musculoskeletal training outcomes. Over the past 25 years, numerous studies have demonstrated its relative safety and efficacy.

**Objectives:**

Blood flow restriction training is under review by the Health Professions Council of South Africa due to safety and ethical concerns. The objective of this roundtable discussion is to gain better insight into the current use and perception of blood flow restriction training in South Africa.

**Formation of panel:**

The expert panel had experience with the use of BFR training and included one representative from each of the following professions, namely, sports medicine, physiotherapy and biokinetics.

**Discussion:**

The panellists provided their unique perspectives on BFR training, whilst reaching a relative consensus on its safety, screening, efficacy, and appropriate use. Agreement on appropriate loading and occlusion pressure protocols during different phases of rehabilitation was less clear.

**Conclusion:**

Although BFR is a safe and effective modality, the development of evidence-based protocols among different health professionals in South Africa is required to ensure good clinical practice.

In July 2022, the Health Professions Council of South Africa (HPCSA) was informed that clinicians in South Africa were being trained to use blood flow restriction (BFR) as a treatment modality. The technique manipulates blood flow and therefore there are some associated ethical and safety concerns. The HPCSA was unable to support the use of BFR due to a lack of submitted information and evidence that allowed its Professional Board for Physiotherapy, Podiatry and Biokinetics (PPB) to make an informed decision. As a result, the HPCSA was tasked to determine if (1) BFR is a valid treatment modality, and (2) whether it fits within the scope of practice of Physiotherapists and Biokineticists in South Africa. At the time of the publication of this roundtable discussion, BFR training had not been incorporated into the toolbox of treatment modalities available to Physiotherapists and Biokineticists in South Africa.

BFR, although practised since the 1970s, was first published in a 1997 study.^[1,2]^ In the subsequent 25 years, a relatively rich body of evidence has been published supporting its use. Despite this, BFR remains a novel technique in South Africa, with little known about how it is used amongst health professionals and what beliefs are held regarding its safety and efficacy. To prepare for its formal submission to the HPCSA and to gain better insight into the use and perception of BFR in South Africa, an expert panel of medical professionals was asked a series of questions. The following panellists were invited to participate: Dr Janesh Ganda, (Sports Physician and Team Doctor of the South African 7s Rugby Team), Lize van Schalkwyk, (Physiotherapist and Head of Physiotherapy at Western Province Rugby and the Stormers Rugby), and David Fabricius, (Biokineticist and Head of Performance and Wellness at the Lambert Sports Clinic, London). Dr Robert Evans (Biokineticist and Clinical Director at the Enable Centre) was the panel facilitator.

Before the various questions were explored, there was a discussion about the definition of BFR to ensure that all panellists were referring to the same techniques and protocols. Following this discussion, the working definition was;

*BFR or occlusion training involves the use of a cuff or tourniquet system placed around the proximal end of a limb and inflated/tightened to a specific pressure (studies range from 110 to 240 mmHg with pneumatic cuffs). Through this pressure, the venous outflow is slowed, whilst arterial flow is maintained – resulting in an anaerobic environment.* ^*[1]*^

Following agreement about the definition, panellists were asked a series of questions.

## Do you consider BFR training safe?

### Janesh Ganda

Yes. Traditionally, when attempting to improve muscle mass and strength, high-intensity resistance training loads of 75%–80% of one repetition maximum (1RM) are often indicated.^[3]^ Using BFR, one can get similar hypertrophic and strength responses at 10%–20% of 1RM. This makes BFR training safe and valuable in the rehabilitation of patients who may not be able to perform high-load resistance training (HL-RT), such as patients undergoing rehabilitation and recovering athletes. BFR enables practitioners to reduce the load on the joint while still placing the limb under appropriate strain.^[3]^

### Lize van Schalkwyk

Yes, I believe it is a safe training modality to use. In a review article, the authors found no significant higher risk in cardiovascular response, venous thromboembolism (VTE), or muscle damage.^[4]^ The principle of BFR training is to work at a resistance level of 20%–40% of 1RM, which will cause less strain on the joints and soft tissue than HL-RT. The percentage of blood flow restriction will be 40%–80% of arterial occlusion pressure (AOP), also known as limb occlusion pressure (LOP), and therefore not causing complete restriction of blood flow. In our clinical setting, we work with elite sportsmen who undergo annual medical screening, including cardiac screening. Our patient population consists of healthy, young athletes. The decision to use BFR training for an injured player is based upon enabling the athlete to start with some form of muscle stimulus and loading at an earlier stage than what is possible with HL-RT. This should therefore also be safe to use in the general population.

### David Fabricius

Yes, BFR with correct implementation and supervision presents no greater risk than traditional modes of exercise, with a low risk of possible adverse responses or concerns of disturbed haemodynamic and ischaemia reperfusion injury. BFR requires an individualised approach when selecting cuff pressure for both safety and effectiveness. Case study reports of adverse responses to acute sessions of BFR, regarding rhabdomyolysis and delayed onset muscle soreness (DOMS), are independent of BFR and include individuals unaccustomed to exercise.^[5]^ Injuries resulting from BFR seem rare and risks of adverse events may be exacerbated in clinical populations (e.g. patients with established cardiovascular disease, hypertension, diabetes mellitus, and chronic kidney disease). Current recommendations suggest 40%–80% of LOP when conducting BFR in clinical populations. Lower pressures may provide less risk without the need for higher pressure. Establishing LOP is quick and easy to perform with a handheld Doppler and may minimise any cardiovascular risk from BFR during exercise, alongside pre-exercise screening.

## What precautions should be taken?

### Janesh Ganda

A pre-participation health screening should be performed, or medical clearance should be attained before initiating BFR. Anatomically, one should be wary of skin, muscle, vessel, and nerve injuries. The main side effects observed by Queiros et al. ^[6]^ were tingling (71%), DOMS (56%) and excessive pain during exercise (45%). Rhabdomyolysis, fainting, and subcutaneous haemorrhage were reported infrequently (1.9%, 3.8%, 4.8% respectively). The main contraindications for the use of the technique are a history of thrombosis and any cardiovascular disorder. Several other contraindications are mostly related to the risk of thrombosis secondary to venous stasis.

### Lize van Schalkwyk

The precautions will have to be standardised in the same manner as applies to all other treatment modalities. It will be important to do a thorough musculoskeletal (MSK) and injury assessment of the patient to determine if and at what stage BFR training will be indicated. The normal precautions for the specific injury will apply. I suggest a standardised health screening that will include any previous pulmonary embolism (PE) or deep vein thrombosis (DVT), family history of PE or DVT, obesity, hypertension, medication, and history of syncope. The practitioner must explain to the patient what BFR will entail, what it feels like, possible lasting sensation changes in post-training, etc. It is important to use the correct pressure for the cuff width and not to leave the patient unattended whilst undergoing BFR training. It is also advisable to ensure there is an emergency action plan in place at the facility in the extreme event of an adverse reaction. This will apply in normal circumstances and not only for BFR training.

### David Fabricius

To ensure that BFR is properly used, and there are efficacy endpoints, it is recommended to use a questionnaire for risk stratification involving a review of the patient’s medical history, and signs and symptoms indicative of any underlying pathology. BFR pre-participation screening theoretically can reduce risks by excluding people with comorbidities or medically complex histories that could unnecessarily heighten the intra- and/or post-exercise occurrence of adverse events. Nascimento et al. proposed a risk stratification tool as a framework to allow clinicians to use their knowledge, skills, and expertise to assess and manage any risks related to the delivery of an appropriate BFR exercise programme.^[7]^

## Do you consider BFR to be an effective training technique?

### Janesh Ganda

BFR has far-reaching effects on multiple body systems, including cardiovascular, pulmonary, vascular, MSK, and the endocrine system. It appears that the greatest benefit of using BFR is its ability to safely augment exercise intensity in both comorbid and healthy individuals. Bond et al.^[8]^ showed successful outcomes in the rehabilitation setting in post-surgical patients (osteochondral fractures, Achilles tendon ruptures, knee arthroscopy); however, caution should be exercised in these patients due to their tendency to also have venous thromboembolism (VTE). An additional benefit of BFR is associated with improving or maintaining VO^2^ max. This is valuable in populations who are unable to exercise at intensities high enough to improve or maintain aerobic capacity.^[9]^

### Lize van Schalkwyk

Yes, I believe BFR training is effective and beneficial. In a systematic review, the authors found changes in both muscle strength and hypertrophy.^[3]^ We started using this technique about four years ago. Some of our patients had underlying joint injuries, i.e. osteochondral knee injuries, patellafemoral dysfunction, etc. and were not able to cope with the normal running load required within HL-RT programmes. These athletes started using BFR during aerobic and/or resistance training as an activation before a field session, and we also adapted their gym programmes to incorporate BFR. Although we did not do any formal research studies, we found strength gains and better functional movement in these athletes.

### David Fabricius

Yes, training with BFR can present beneficial adaptations to skeletal muscle strength, muscle mass, and performance in different population groups when combined with strength and aerobic training. Low-load resistance training (LL-RT) with BFR (20%–30% of 1RM) may be superior at increasing muscle strength and mass to that of LL-RT alone, and comparable to HL-RT without BFR (70%–80% of 1RM) in people with MSK conditions. If increasing strength is the aim of the training and heavy loads cannot be tolerated or are contraindicated, then LL-RT with BFR training is an evidence-based option. In addition to resistance training, BFR has been shown to improve muscle performance and aerobic capacity during aerobic exercise (~45% VO^2^max) and can be performed passively to prevent muscle atrophy and improve physical function.

## In what situations may it be beneficial?

### Janesh Ganda

BFR has widespread applications, including being incorporated into training for high-level athletes or post-operative rehabilitation for patients with limited activity and the ability to undertake weight-bearing exercises. This includes patients who have suffered spinal cord injuries. Given the ability of BFR to stimulate gains at a submaximal load, athletes can often incorporate this sort of treatment at the end of their workout to achieve greater strength gains. BFR training can lead to significant improvements in muscle strength, markers of sports performance and muscle size.^[1]^

### Lize van Schalkwyk

We use BFR on all patients that have undergone lower limb surgery and must be immobilised for a period, who cannot weight-bear post-surgery, and when loaded gym work is still contraindicated. We combine it with neuromuscular electrical stimulation during open chain exercises – either focusing on the quadriceps, hamstrings, or gastrocnemius muscles. Examples of injuries where we have used BFR include, anterior cruciate ligament (ACL) reconstruction, microfracture surgery for femoral and patella osteochondral lesions, Achilles rupture and syndesmosis repair. We have recently started using BFR on the upper limb after shoulder surgery. As mentioned previously, we find BFR beneficil for athletes who are unable to cope with standard training loads.

### David Fabricius

As a novel method of exercise training, BFR could be used as a safe alternative to HL-RT with broad applications in clinical populations, the elderly, rehabilitation after injury or post-surgery and healthy athletic populations.^[1]^ Multiple benefits exist beyond that of muscular growth, including improvement in muscular endurance, cardiovascular fitness, pain, and bone density. It is well documented that muscle hypertrophy and strength adaptations with BFR are significantly greater than those achieved with LL-RT alone. Such adaptations have been observed after only one to three weeks.

## How and where should BFR be used by health professionals in South Africa?

### Janesh Ganda

The implementation of BFR training requires a BFR device, which can range from an inexpensive “wrap”, a regular pneumatic manual cuff, or an automated cuff. The gold standard to determine cuff pressure is the use of AOP which requires the use of Doppler Ultrasound to determine the pressure required to cease blood flow to the limb. Percentage of resting systolic blood pressure (SBP) can also be used. Recommendations include 80% resting SBP for continuous BFR training and 130% for intermittent training. Currently prescribed protocols for resistance training include an intensity of 20%–30% of 1RM, short intervals, and a volume of up to 75 repetitions, which can be divided into four sets of 30/15/15/15 repetitions respectively.^[4]^ Little equipment is required for the implementation of BFR training. It should therefore be carried out in a rehabilitation or clinical practice setting with the treating practitioners having at least a basic knowledge of physiology and anatomy to ensure patients do not have any contraindications.

### Lize van Schalkwyk

BFR training is currently not widely used in South Africa. I have not come across peers that use it in their clinical environment. I believe there is scope within the physiotherapy regimes to use passive BFR for patients that are immobilised for long periods of time due to paralysis, illness, surgery, etc. BFR aerobic and resistance exercise can also be used to prevent muscle atrophy and maintain function both pre- and post-surgery or post-injury when higher load training is still contraindicated. BFR must be used under supervision, with the principles and possible side effects explained to the patient. The therapist and patient should sign a consent form, as is the case with most treatment modalities.

### David Fabricius

**Fig. 1:**
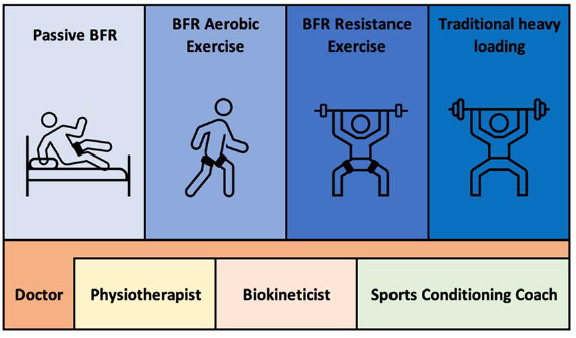
Progressive multidisciplinary BFR rehabilitation model ^[11]^

In the South African context, the physical rehabilitation process is often shared by practitioners from different disciplines. The decision about which practitioner is best suited depends on their scope of practice and the timeframe of the injury (Figure 1). BFR is not a stand-alone modality to improve outcomes for a specific diagnosis or condition, but rather a useful adjunct. The simple addition of a BFR cuff to LL-RT under the supervision of a competent professional will lead to achieving superior results in muscle adaptations or functional capability. The suggested multidisciplinary, staged approach in Figure 1 corroborates the evidence-based progressive model of Loenneke et al. ^[10]^ This approach consists of four phases: (1) passive BFR; (2) BFR with aerobic training; (3) BFR with LL-RT; and (4) BFR with low-load LL-RT in combination with traditional HL-RT.^[11]^ These phases can be integrated into the stages of a traditional MSK rehabilitation programme.

## Which health profession’s scope of practice does BFR fall within?

### Janesh Ganda

BFR training should fall within the scope of practice of Physiotherapists and Biokineticists in South Africa. This is due to their role in rehabilitation following acute injuries and the knowledge of physiology and anatomy ensuring that patients safely meet the criteria for BFR training.

### Lize van Schalkwyk

There must be a distinction made between using BFR as a modality for an injured person versus using it for the general population as a training modality. In the medical environment, the referring doctor can prescribe BFR as part of the treatment protocol. If a physiotherapist is treating the patient, he/she should inform the referring doctor of the intent to use the modality and clarify any potential risk factors with the doctor. The same will apply to a biokineticist who is treating a patient that was referred to them. There must be a standardised protocol i.e. consent, risk factors, contraindications, etc. In our facility, it will be a joint decision between the physiotherapist and the strength and conditioning trainers.

### David Fabricius

The doctor and physiotherapist are entitled to screen and prescribe both the passive and active applications of BFR within rehabilitation. Both the doctor and physiotherapist are likely to address the early assessment and treatment within both in- and out-patient capacities. Biokineticists can screen and utilise BFR where active movement is indicated. In reference to Figure 1, biokineticists can start treatment from Stage Two onwards (BFR aerobic exercise) and can facilitate rehabilitation or training up to Stage Four (traditional heavy loading). Physiotherapists can complete the entire rehabilitation process. However, within any multidisciplinary setting, each practitioner plays a pivotal and collaborative role in the envisioned rehabilitation process.

## What do you envision the future of BFR training to be?

### Janesh Ganda

The ease of application and benefits of BFR training should make this modality of training more popular, specifically in athletes recovering from injury, post-surgical patients, and the elderly. The MSK benefits (increase in muscle size and strength) which can be achieved at a fraction of the 1RM reduce the loading on the joint while still achieving the benefits of resistance training.

### Lize van Schalkwyk

BFR is currently not widely used in the clinical environment. There is a large body of research published that will help to standardise protocols. Numerous studies use different cuff sizes, different pressures, different loads, and rest periods, but they all appear to achieve positive results in increasing muscle strength and hypertrophy. As more clinicians start using BFR, there will be better study outcomes to support its benefits.

### David Fabricius

Within the available literature, great strides have been made in utilising BFR in the treatment areas of neurological conditions, MSK conditions and physical performance enhancement in athletes. As understanding grows behind the mechanisms and physiology of BFR, this field of interest will broaden but give way to more specific protocols and guidelines of treatment.

## Conclusion

This roundtable discussion demonstrates relative consensus on numerous fundamental elements of BFR, including its safety, screening, efficacy and appropriate use amongst health professionals in South Africa. What is less clear are the relative loading and occlusion pressure protocols in the different phases of rehabilitation. BFR is a relatively new modality and detailed knowledge of its application is lacking. It follows that practitioners should be focused on their duty of care to screen and prescribe BFR effectively and safely. Standardised training and the development of evidence-based protocols across different health professionals are required to ensure good clinical practice. The rapid dissemination of such skills to our health professionals in South Africa will provide a novel and effective tool which may contribute to bridging the acute and longer-term rehabilitation of numerous patients.
